# Optimization of parameters for femoral component implantation during TKA using finite element analysis and orthogonal array testing

**DOI:** 10.1186/s13018-018-0891-1

**Published:** 2018-07-20

**Authors:** Zhifang Mou, Wanpeng Dong, Zhen Zhang, Aohan Wang, Guanghong Hu, Bing Wang, Yuefu Dong

**Affiliations:** 1 Department of Critical Care Medicine, The Affiliated Lianyungang Hospital of Xuzhou Medical University/the First People’s Hospital of Lianyungang, Lianyungang, China; 2 School of Materials Engineering, Shanghai University of Engineering Science, Shanghai, China; 3 Institute of Plasticity Forming Technology & Equipment, Shanghai Jiao Tong University, Shanghai, China; 4 Department of Orthopedics, The Affiliated Lianyungang Hospital of Xuzhou Medical University/the First People’s Hospital of Lianyungang, Lianyungang, China

**Keywords:** Total knee arthroplasty (TKA), Prosthesis, Implantation parameter, Optimization, Finite element analysis, Orthogonal array testing

## Abstract

**Background:**

Individualized and accurate implantation of a femoral component during total knee arthroplasty (TKA) is essential in achieving equal distribution of intra-articular stress and long-term survival of the prosthesis. However, individualized component implantation remains challenging. This study aimed to optimize and individualize the positioning parameters of a femoral component in order to facilitate its accurate implantation.

**Methods:**

Using computer-simulated TKA, the positioning parameters of a femoral component were optimized individually by finite element analysis in combination with orthogonal array testing. Flexion angle, valgus angle, and external rotation angle were optimized in order to reduce the peak value of the pressure on the polyethylene liner of the prosthesis.

**Results:**

The optimal implantation parameters of the femoral component were as follows: 1° flexion, 5° valgus angle, and 4° external rotation. Under these conditions, the peak value of the pressure on the polyethylene liner surface was minimized to 16.46 MPa. Among the three parameters, the external rotation angle had the greatest effect on the pressure, followed by the valgus angle and the flexion angle.

**Conclusion:**

Finite element analysis in combination with orthogonal array testing can optimize the implantation parameters of a femoral component for TKA. This approach would possibly reduce the wear of the polyethylene liner and prolong the survival of the TKA prosthesis, due to its capacity to minimize stress. This technique represents a new method for preoperative optimization of the implantation parameters that can achieve the best possible TKA outcome.

## Background

Total knee arthroplasty (TKA) is an effective therapy for terminal-stage osteoarthritis of the knee and can ameliorate pain, correct deformity, and improve the function of the joint [1]. Over 90% of implanted prostheses last approximately 10 to 15 years following TKA [2–4]. To date, TKA is a proven technique that is based on established surgical principles, among which accurate osteotomy and prosthesis implantation are fundamental requirements. TKA aims to restore the neutral mechanical alignment of the lower extremity and promote the uniform distribution of stress in the knee joint, thereby prolonging the survival of the prosthesis by reducing the wear on the polyethylene liner [5, 6]. Although a great number of factors can influence the survival of an implanted prosthesis, the surgical error that leads to implant malalignment is the most common cause of TKA failure [7–13]. The consequences of implant malalignment include an uneven distribution of intra-articular load and of stress that eventually will require a revision [14]. Accurate implantation of an individualized TKA prosthesis could effectively reduce the surgical error and avoid the uneven distribution of the intra-articular load, thereby stabilizing the knee joint, reducing prosthetic loosening, and improving knee function [15–18].

The implantation of a TKA prosthesis aims to optimally restore the neutral mechanical alignment of the lower extremity, improve the therapeutic effect of the TKA, and prolong the survival of the prosthesis. Currently, the parameters used to guide the implantation of the prosthesis during TKA are usually determined by image data of the lower extremity [19]. Computer-navigated TKA can improve the accuracy of TKA prosthesis implantation and achieve a postoperative lower limb mechanical axis that is closer to the ideal position [20, 21]. Furthermore, patient-specific instrumentation has been reported to confer advantages over traditional TKA techniques with regard to the accuracy of prosthesis implantation, the efficient restoration of the neutral mechanical axis of the lower extremity, and the extension of the prosthesis survival [22, 23]. However, although a preoperative plan that is based on the anatomic features of a patient can aid the restoration of the neutral mechanical axis of the lower extremity, it cannot directly reveal the distribution of intra-articular stress and/or reliably predict the survival of the TKA prosthesis.

In the case of evenly distributed intra-articular stress, the contact surface area is increased and the stress on the polyethylene liner is decreased. This can maintain the long-term survival of the prosthesis. Therefore, an optimization of the prosthesis implantation parameters that is based on the intra-articular stress distribution in an individual patient could cause the even distribution of intra-articular stress following TKA. This strategy would be more efficient compared with a conventional preoperative planning. However, how to effectively determine the individualized optimal implantation parameters before TKA to make sure that stress of the knee joint is evenly distributed and the value is the smallest, as well as clarify the influence of different implantation parameters on stress to guide the accurate implantation of prosthesis during TKA surgery is currently a difficult problem of preoperative plan of TKA. Finite element analysis is an effective method for optimizing prosthesis implantation parameters that has been widely utilized for the design, selection, and postoperative evaluation of TKA prostheses [24, 25]. The repeatability of a finite element model allows the implantation parameters of TKA prosthesis to be effectively optimized. Using the finite element method, Cheng et al. analyzed the surface stress on the polyethylene liner caused by medial and lateral translation, anterior and posterior translation, and external rotation [26]. This study demonstrated that the peak value of the contact stress was altered by these positioning changes, with external rotation having the greatest effect [26]. Similarly, Kang et al. highlighted that the increase in the external rotation angle resulted in a higher peak value of contact stress [13]. Although these studies demonstrated the potential clinical utility of finite element analysis for the optimization of TKA, this method has not yet been utilized to guide the implantation of an individualized TKA prosthesis. To date, a limited number of studies have described the use of finite element models in the preoperative planning of an individualized TKA prosthesis for implantation. Maybe this is due to the numerous TKA finite element models that require an extensive calculation time, which in turn limits the clinical application of this approach.

Orthogonal array testing can effectively reduce the number of TKA finite element models and improve the efficiency of the analysis prior to surgery. However, the use of orthogonal array testing for finite element modeling of TKA prosthesis has not been previously reported. We hypothesized that the implantation parameters of the femoral component could be optimized using a finite element model of the knee joint in combination with orthogonal array testing, thereby reducing the peak value of the pressure on the polyethylene liner and decreasing wear. Therefore, the main objectives of our study were (1) to determine whether finite element analysis in combination with orthogonal array testing could be used to reduce the stress on the polyethylene liner, (2) to obtain the ideal optimization, and (3) to establish which implantation parameter had the most influence on stress distribution. It was anticipated that optimization of the implantation parameters using finite element analysis and orthogonal array testing, based on the anatomy of each individual patient, could be used clinically as part of individualized preoperative planning to improve the accuracy of TKA prosthesis implantation.

## Methods

### Finite element model of the TKA knee joint

#### Three-dimensional (3D) model of the TKA prosthesis

**Fig. 1:**
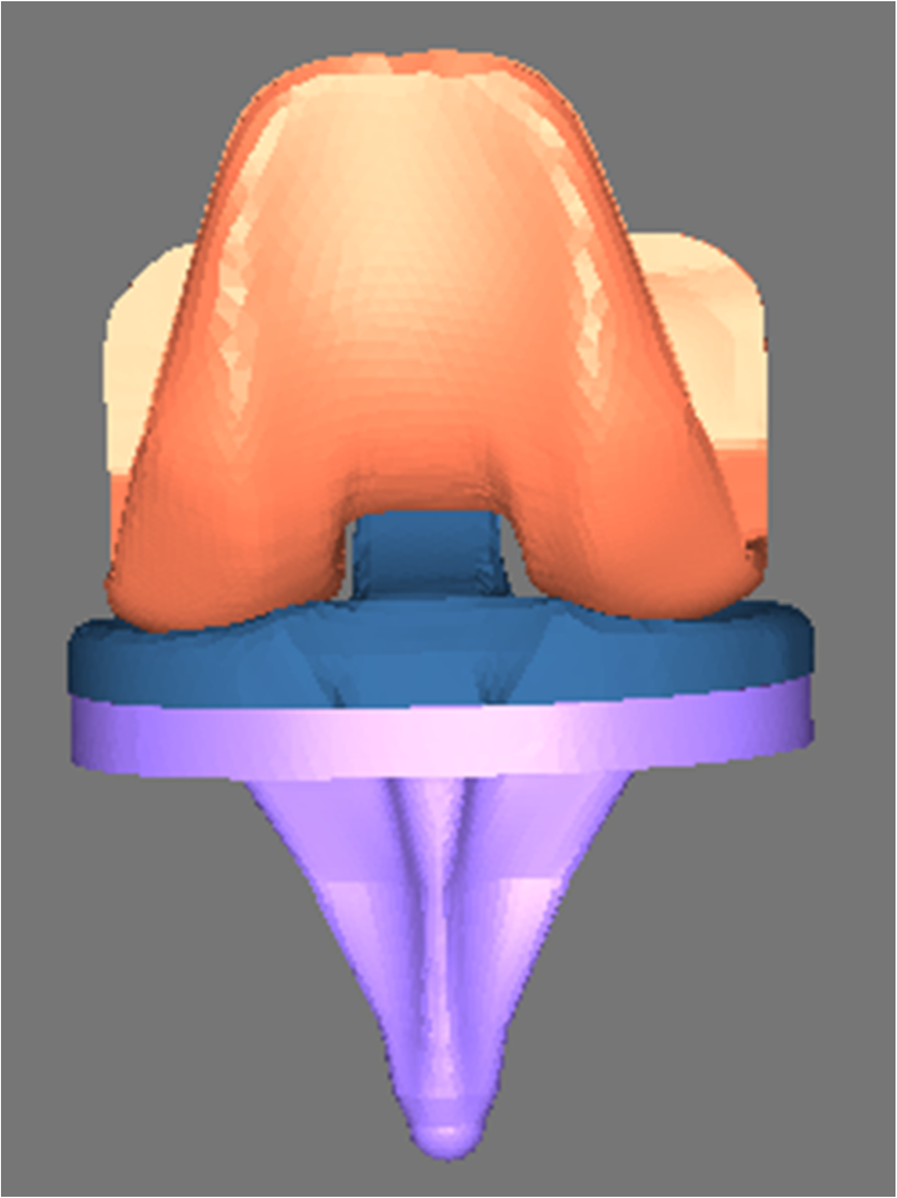
3D model of the components of the prosthesis used for simulated TKA

The present study was approved by our institutional review board (No. 2016008), and informed consent was obtained from the volunteer. A cemented, posterior-stabilized knee prosthesis system (Smith and Nephew, Memphis, TN, USA) was scanned by the IMS IMPAC laser scanner (Renishaw, London, UK), and the .stl files were acquired. The .stl files were imported to the Mimics 12.0 software (Materialise, Leuven, Belgium), and 3D models of the TKA prosthetic components were generated (Fig. 1).

#### Simulated surgical implantation of the TKA prosthesis

**Fig. 2:**
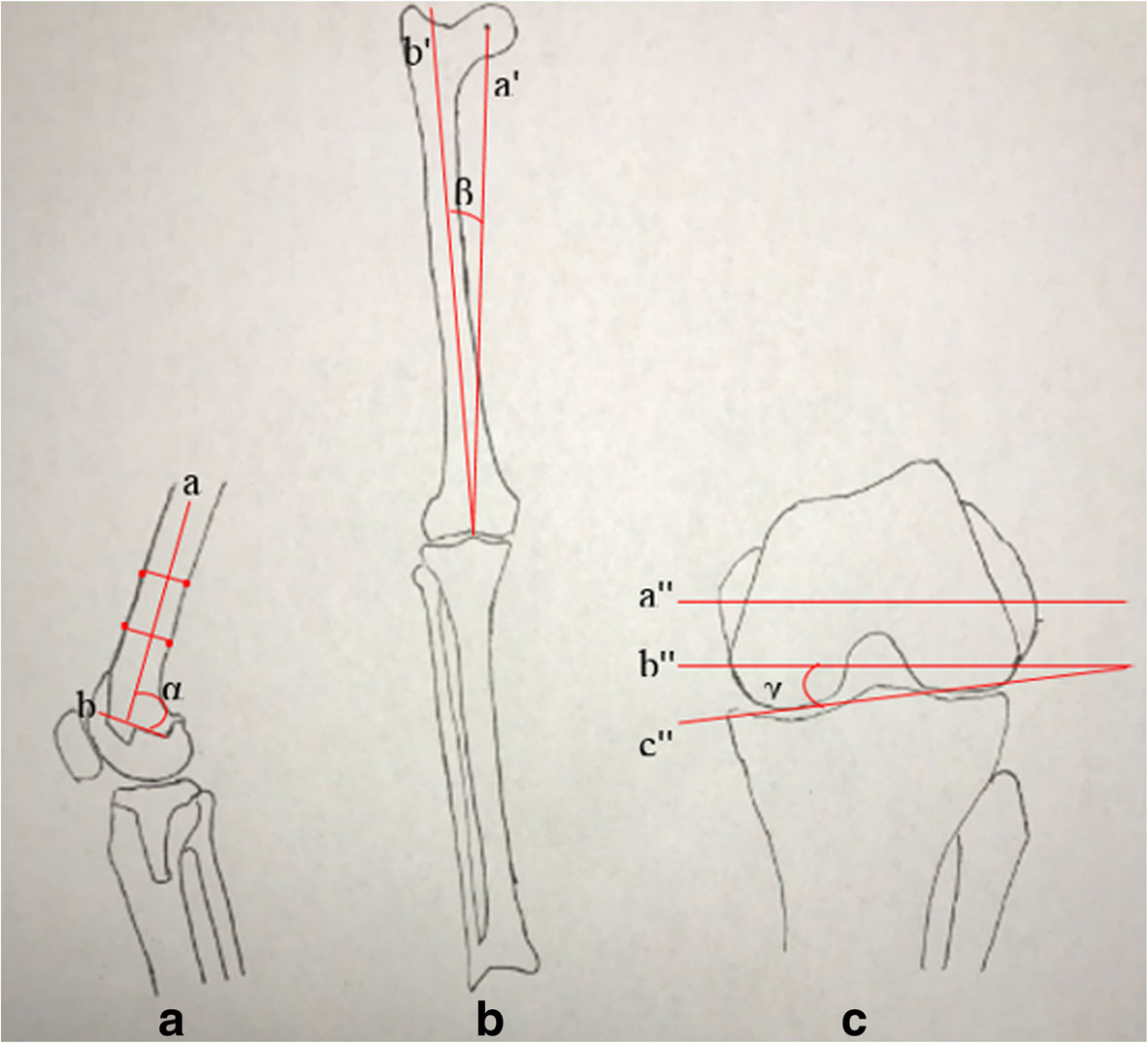
Diagram illustrating the measurement of the implantation parameters for the TKA femoral component. **a** The flexion angle (in the sagittal plane) of the femoral component (α angle). Line a is the femoral shaft axis; line b lies along the bottom of the femoral implant. **b** The valgus angle (in the coronal plane) of the femoral component (β angle). Line a’ is the anatomic axis of the femur; line b’ is the mechanical axis of the femur. **c** The external rotation angle (in the axial plane) of the femoral component (γ angle). Line a” is the transepicondylar axis; line b” is parallel to the transepicondylar axis; and line c” is the posterior condylar line of the femur

**Fig. 3:**
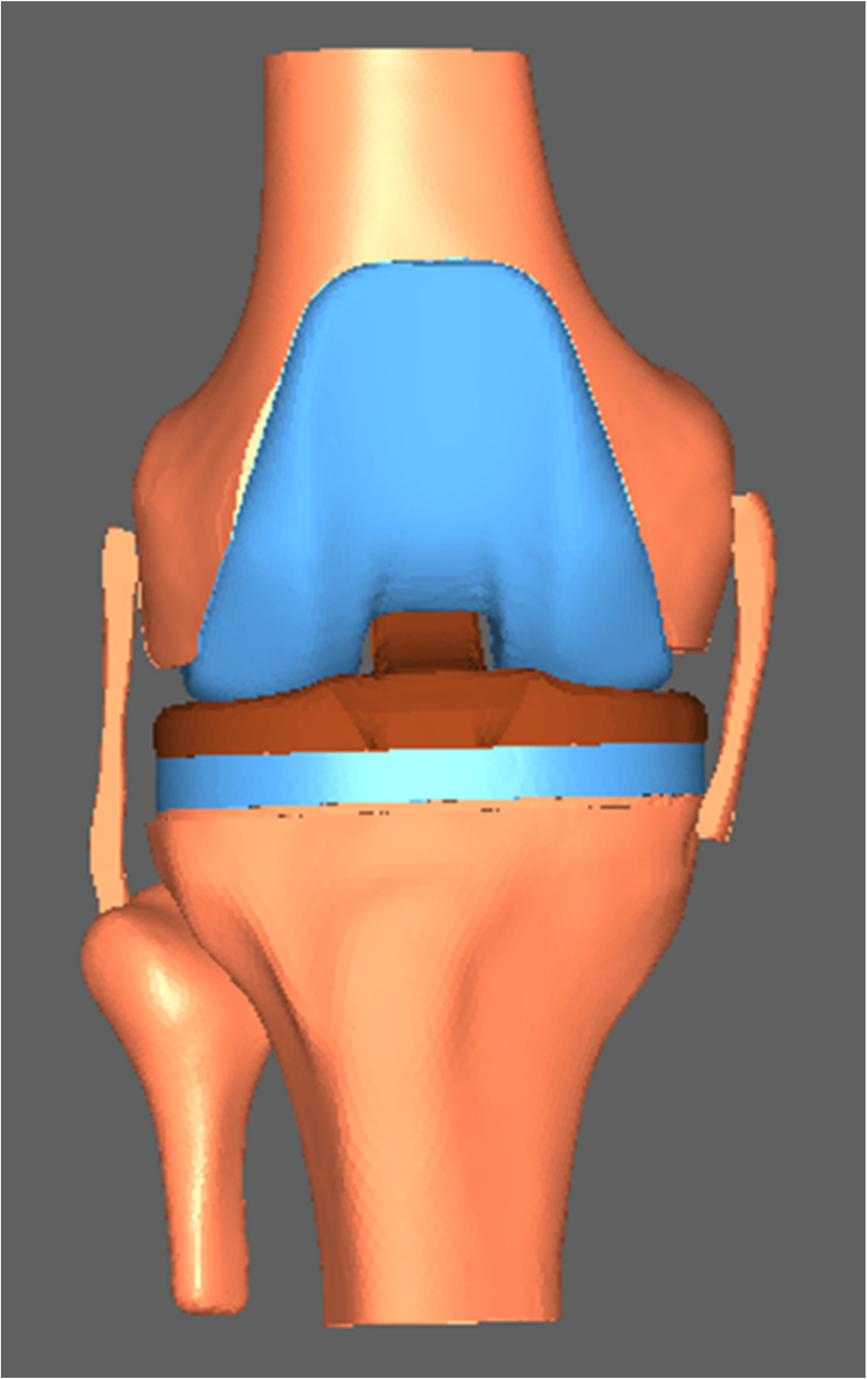
3D model of the TKA knee

The 3D models of the knee joint that were generated and validated by our previous studies were implanted by simulation surgery [27, 28]. Long-leg weight-bearing radiographs were obtained from a 24-year-old man. Based on the anatomic characteristics of these radiographs, the valgus angle of the anatomic axis of the femur (i.e., relative to the long femoral shaft) and the mechanical axis of the femur (i.e., relative to the line connecting the center of the femoral head and the center of the knee joint) was 6°. Thus, the valgus angle of the distal femur was 6°. The femoral component implantation parameters were determined according to the conventional principles of TKA surgery: flexion 0° (i.e., the distal end of the femoral component was perpendicular to the femoral shaft axis in the sagittal plane), valgus 6° (in the coronal plane), and external rotation 3° (i.e., the posterior condylar line was rotated 3° externally with respect to the transepicondylar axis in the axial plane) (Fig. 2). These parameters were regarded as the standard parameters for positioning of the implanted femoral component and were used to construct the standard TKA model. Based on the anatomic characteristics of the lower extremities and knee joints of the volunteer as well as the principles of TKA surgery, an osteotomy was performed on the model of the femur and tibia using Mimics software. Based on the measurements obtained, No. 5 femoral and tibial component, 9-mm polyethylene liners, and 1-mm osteotomy plate were used to perform the osteotomy. A 1-mm gap was included between the prosthesis and osteotomy surface to preserve a space for implantation of a 1-mm-thick bone cement layer. First, a distal femoral osteotomy was performed. The osteotomy surface was perpendicular to the femoral mechanical axis in the coronal plane and perpendicular to the femoral shaft axis in the sagittal plane so as to ensure a neutral implantation in the coronal and sagittal planes. The resection thickness was 11 mm to the distal articular surface of the medial condyle. The posterior condylar line was rotated 3° externally with respect to the transepicondylar axis in the axial plane. The anterior and posterior cut and the anterior and posterior oblique cut of the distal femur were obtained to complete the femoral osteotomy. Subsequently, a proximal tibial osteotomy was performed. The osteotomy surface was perpendicular to the tibial mechanical axis in the coronal plane with a posterior slope of 5° in the sagittal plane so as to ensure a neutral implantation in the coronal and sagittal planes. The thickness of the resection was 10 mm to the highest point of the lateral tibial plateau. The central line of the tibial component was aligned to the medial 1/3 of the tibial tubercle in the axial plane to complete the proximal tibial osteotomy. Finally, the femoral component, tibial component, and polyethylene liner were implanted to obtain a three-dimensional model of the knee joint after TKA (Fig. 3). After implanting the TKA prosthesis, two-dimensional and three-dimensional measurements and observations were used to confirm that the tangent surface of the most posteriorly edges of the femoral component and tibial component was aligned vertically. Posteriorly, the centerline of the femoral component was aligned to the centerline of the tibial component. Additionally, the contact areas between the femoral component and the medial and lateral compartments of the polyethylene liner were maximal and equal, ensuring matching between the components.

#### Finite element model of the TKA knee

**Fig. 4:**
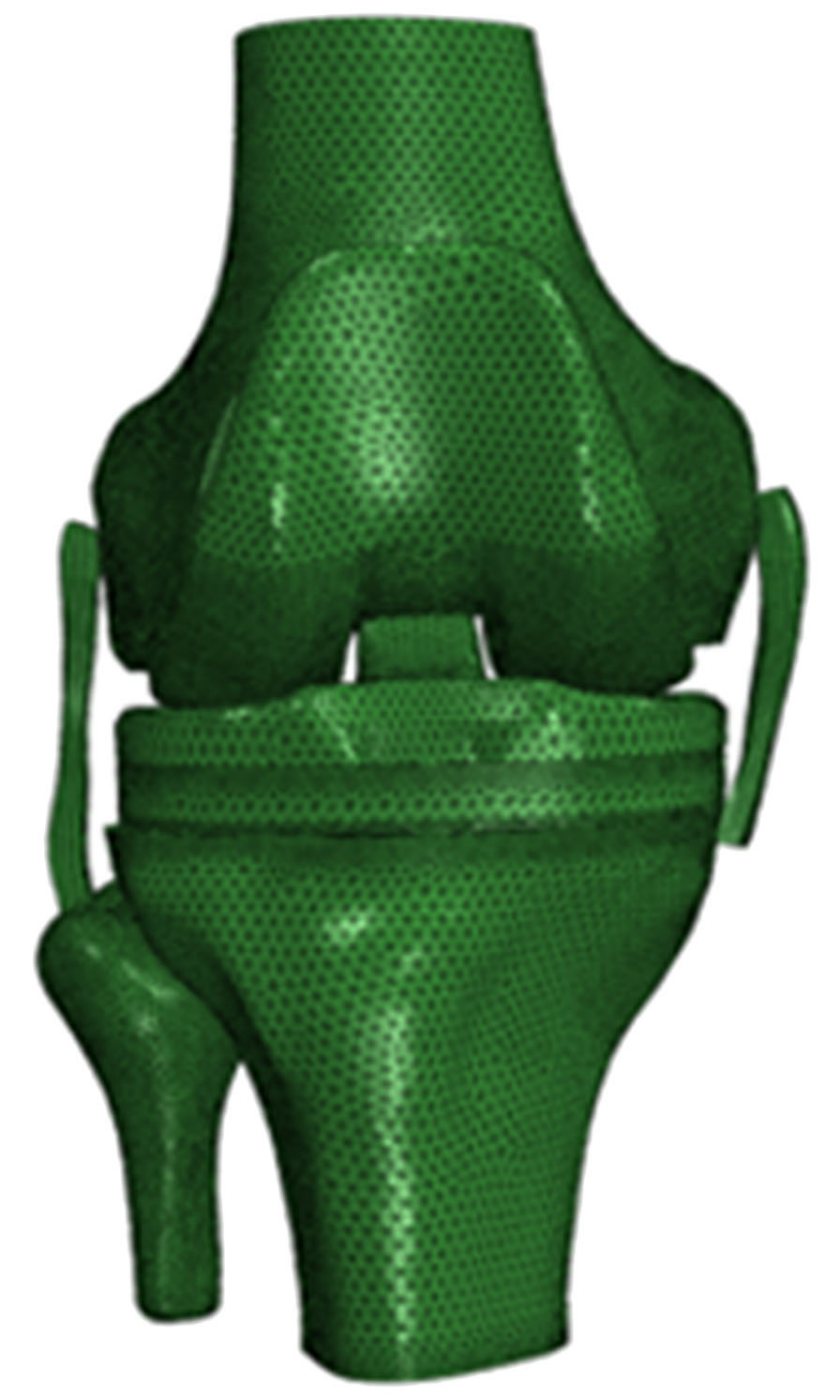
3D finite element model of the TKA knee

The 3D models of the knee anatomic structures and TKA prosthetic components were imported into Hypermesh 15.0 software (Altair, Clifton Park, NY, USA) as .stl files; the space between the femoral component and the distal end of the femur and the space between the tibial component and the proximal end of the tibia were filled with 1-mm-thick bone cement [29]. Following generation of the mesh, the 3D finite element model of the TKA prosthesis, which we termed the standard model of the TKA knee joint, was constructed to include the femur, tibia, fibula, medial, and lateral collateral ligaments, as well as the femoral component, tibial component, polyethylene liner, and bone cement layers (Fig. 4).

#### Material properties, boundary conditions, and loading

The finite element analysis was carried out using the general-purpose FE code Abaqus v.6.14 (Simulia, Providence, RI, USA). The ligaments were defined as anisotropic and hyperelastic and were modeled by an incompressible Neo-Hookean behavior with energy density function: $\psi ={C}_1\left(\overset{\sim }{I_1}-3\right)$(1), where *C*_1_ is the initial shear modulus, and $\overset{\sim }{I_1}$ is the first modified invariant for the right Cauchy-Green strain tensor [30]. The *C*_1_ values of the lateral collateral ligament and medial collateral ligament were defined as 6.06 and 6.43, respectively [31]. The number of elements and the number of nodes were (964, 1620) and (1313, 2062), respectively. The remaining anatomic structures and TKA prosthetic components were modeled as isotropic linear elastic materials (Table 1). The following boundary conditions were defined:

1. The femur was limited at 0° flexion position, and all other two rotations and three translations were unconstrained. The tibia and fibula were limited in all translations and rotations.
2. Binding constraints were defined between the cortical bone and the cancellous bone, the prosthesis and the bone cement layer, and the bone cement layer and the osteotomy surface [32].
3. The lateral and medial collateral ligaments were rigidly attached to their corresponding bones, which facilitated the modeling of the ligament-bone attachment.
4. Nonlinear contact with a friction coefficient of 0.04 was assumed for the contact surfaces [33]. Two contact pairs were generated: one between the femoral component and polyethylene liner, and the other between the polyethylene liner and tibial component. The contact conditions were set as small sliding and finite sliding.

**Table 1:** Material properties, element number, and node for the TKA knee

	Elastic modulus (MPa)	Poisson’s ratio	Element number	Node number
Cortical bone	16,600	0.3	23,117	7865
Cancellous bone	2400	0.3	53,675	12,024
Femoral component	210,000	0.3	84,796	21,431
Tibial component	117,000	0.3	37,532	9551
Polyethylene liner	685	0.4	28,248	7917
Bone cement layer	3000	0.3	32,852	11,144

A reference vertical compressive load of 1150 N (along the *Z* axis, approximately twice the body weight) was applied to the midpoint of the femoral transepicondylar axis, simulating the load of the gait cycle for the 0° flexion position [27, 28, 30]. The variable in the peak value of the pressure on the polyethylene liner was observed.

### Orthogonal array testing and optimization analysis

Orthogonal array testing is a design method that uses orthogonal tables to arrange and analyze multi-factor experiments. Orthogonal array testing selects some representative combinations from all the combinations of the experimental factors to perform the experiments. Through analysis of the results of these experiments, an optimal combination of experimental factors can be determined in a highly efficient and time-saving manner. Furthermore, through range analysis, the influence of various factors on the experimental indicators can be obtained, and the order of these factors can be determined. The advantages of orthogonal array testing and range analysis include (1) by taking advantage of the obtained experimental data, correct conclusions can be drawn based on a small number of tests rather than a comprehensive series of tests, thereby saving time; (2) the goal of optimization can be achieved; (3) the influence of various factors on the experimental indictors can be quantified; and (4) it is a straightforward technique to use, only requiring the arrangement of the experimental combinations according to the orthogonal tables.

The parameters flexion angle (A), valgus angle (B), and rotation angle (C) that were related to the positioning of the implanted femoral component were selected for optimization. Three levels were specified for each factor: the flexion angle of the femoral component was set at 0°, 1°, or 2°; the valgus angle was set at 5°, 6°, or 7°; and the rotation angle was set at 3°, 4°, or 5°. An orthogonal table, L_9_(3^4^), was selected (Table 2). The standard TKA model was re-adjusted in Hypermesh software to construct different TKA models that fulfilled the requirement of the orthogonal array testing.

**Table 2:** Design of the orthogonal array testing

Plane	Experimental factors		
	Flexion angle A (°)	Valgus angle B (°)	External rotation C (°)
1	0	5	3
2	1	6	4
3	2	7	5

According to the orthogonal array testing, nine TKA finite element models were generated (using different combinations of position parameters of the femoral component). The definition of the material properties, boundary conditions, and loading was identical for each model. The peak values of the pressure induced by different implantation parameters were compared and ranked using finite element analysis and orthogonal array testing. Subsequently, the minimal peak value of the pressure was obtained, and the corresponding implantation parameters were considered to be the optimal parameters of the femoral component.

### Validation of the optimized parameters derived from the orthogonal array testing

**Fig. 5:**
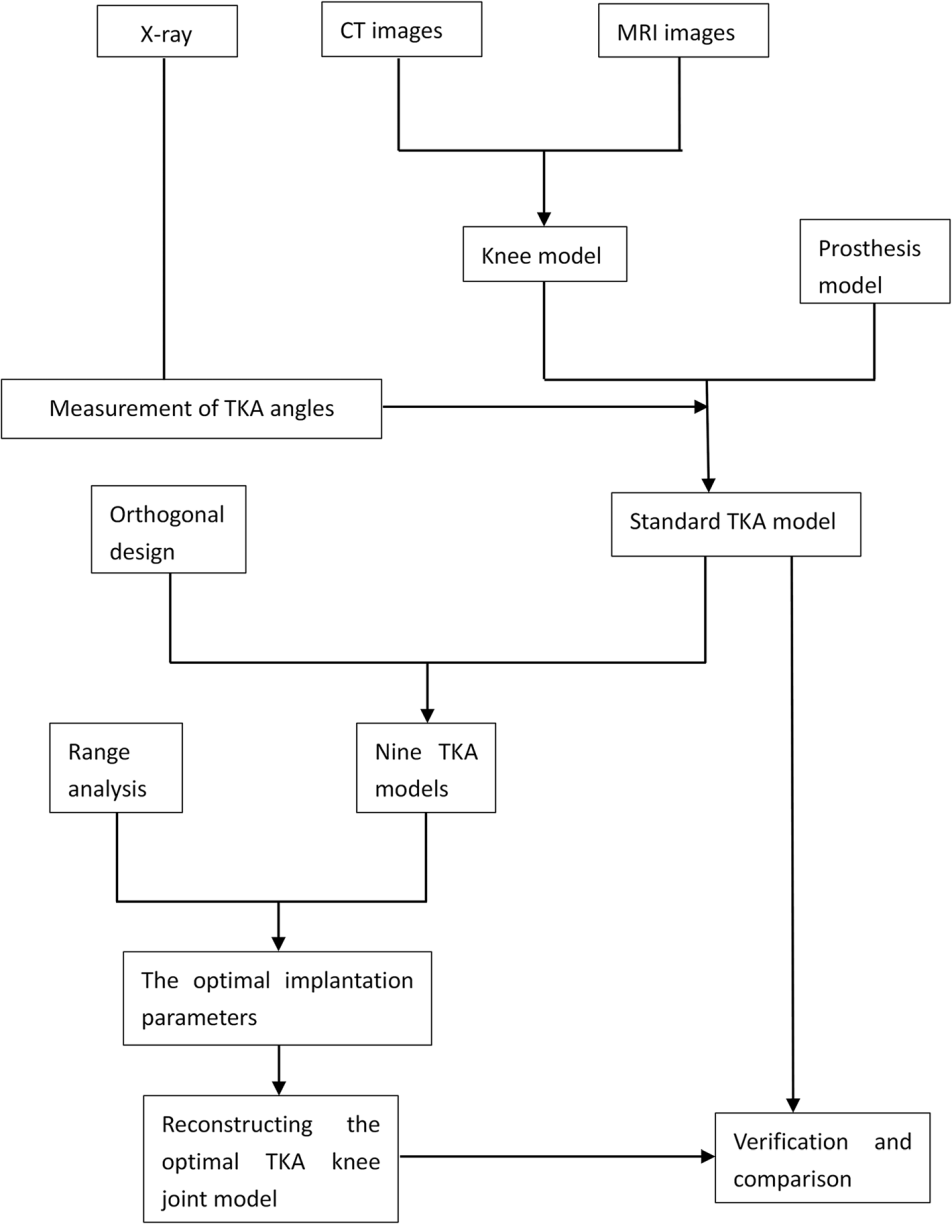
Construction of the TKA knee joint model and its optimization using orthogonal array testing

A finite element model of the TKA knee was re-constructed using the implantation parameters optimized by the orthogonal array testing. The distribution of the peak pressure of the pressure on the polyethylene liner was analyzed and compared with that of all the other models in order to validate the optimized parameters (Fig. 5).

## Results

### Distribution of the pressure on the polyethylene liner

**Fig. 6:**
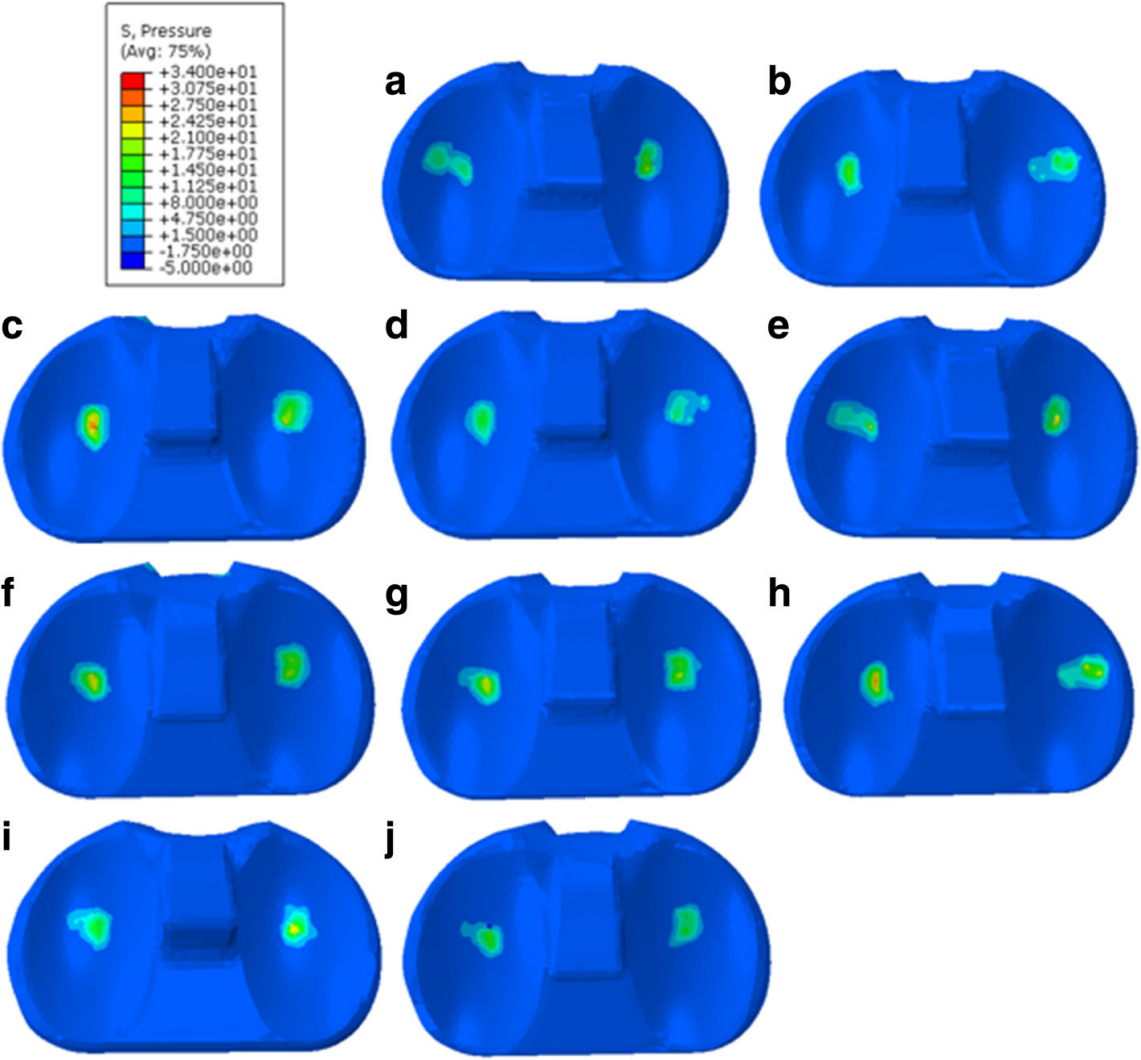
Distribution of the pressure on the polyethylene liner. **a**: model of group A_1_B_1_C_1_. **b**: model of group A_1_B_2_C_2_. **c**: model of group A_1_B_3_C_3_. **d**: model of group A_2_B_1_C_2_. **e**: model of group A_2_B_2_C_3_. **f**: model of group A_2_B_3_C_1_. **g**: model of group A_3_B_1_C_3_. **h**: model of group A_3_B_2_C_1_. **i**: model of group A_3_B_3_C_2_. **j**: standard model

The distribution of the pressure on the polyethylene liner was acquired, and the peak value of the stress for each model is shown in Table 3. The highest peak value of the pressure among the nine models was 30.83 MPa (group A_1_B_3_C_3_, Fig. 6c), which occurred in the medial compartment; the lowest peak value of the pressure was 16.46 MPa (group A_2_B_1_C_2_, Fig. 6d), which was also observed in the medial compartment. The findings indicated that minor variations in the implantation parameters could result in relatively large changes in the stress, highlighting the importance of accurate prosthesis implantation. The standard model of the TKA knee was not included in the nine constructed models but was also examined: the peak value of the pressure on the polyethylene liner was 18.14 MPa (Fig. 6j) and was localized in the medial compartment.

**Table 3:** Experimental level combinations of the orthogonal array testing

Model	Experimental level combination	Flexion angle (°)	Valgus angle (°)	External rotation (°)	Peak value of the pressure (MPa)
1	A_1_B_1_C_1_	0	5	3	21.29
2	A_1_B_2_B_2_	0	6	4	19.82
3	A_1_B_3_C_3_	0	7	5	30.83
4	A_2_B_1_C_2_	1	5	4	16.46
5	A_2_B_2_C_3_	1	6	5	27.25
6	A_2_B_3_C_1_	1	7	3	24.49
7	A_3_B_1_C_3_	2	5	5	24.07
8	A_3_B_2_C_1_	2	6	3	30.22
9	A_3_B_3_C_2_	2	7	4	23.68

### Parameter optimization by orthogonal array testing

Using the orthogonal array experimental design, the range analysis of the peak values of the pressure from the nine models was carried out. The results of this analysis are shown in Table 4, in which *K*_*ji*_ represents the mean of the peak value of the stress for the experimental factor *j* at level *i*.

**Table 4:** Optimization of the results of the orthogonal array testing

Model	Experimental factor *j* (*j* = 1, 2, 3, 4)				Peak value of the pressure (MPa)
	A	B	C	D	
1	1	1	1	1	21.29
2	1	2	2	2	19.82
3	1	3	3	3	30.83
4	2	1	2	3	16.46
5	2	2	3	1	27.25
6	2	3	1	2	24.49
7	3	1	3	2	24.07
8	3	2	1	3	30.22
9	3	3	2	1	23.68
*K*_*j*1_	23.98	20.61	25.33	24.07	
*K*_*j*2_	22.73	25.76	19.99	22.79	
*K*_*j*3_	25.99	26.33	27.38	25.84	
Rang *R*_*j*_	3.26	5.72	7.39	3.05	
Ranking	C > B > A				
Optimal level	A_2_	B_1_	C_2_		

The range was calculated using the following formula: ${R}_j=\underset{1\le i\le m}{\max }{k}_{ji}-\underset{1\le i\le m}{\min }{k}_{ji}$ (2), where *R*_*j*_ represents the range of experimental factor *j*, and *m* represents the number of the level. For example, the maximum *K*_13_ was 25.99 and the minimum *K*_12_ was 22.73 under the first factor; thus, the range *R*_1_ (*K*_13_-*K*_12_) was 3.26. The range analysis suggested that among the three factors studied, the external rotation angle exhibited the highest pressure effect on the polyethylene liner, followed by the valgus angle and the flexion angle.

**Fig. 7:**
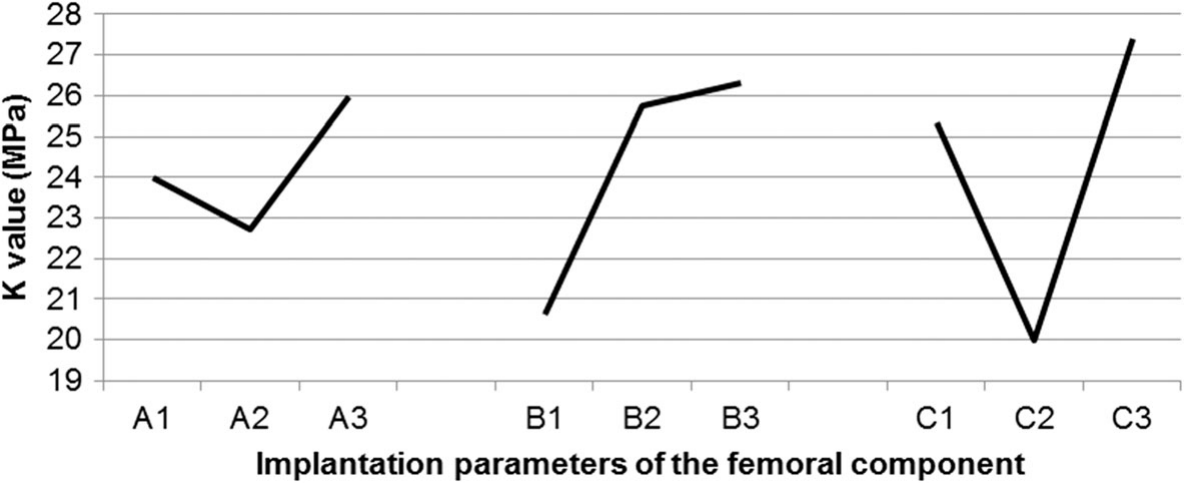
Trend diagram showing the variations in the peak value of the pressure (*K* value) caused by changes in the implantation parameters of the femoral component

A trend diagram indicating the variations in the peak value of the stress that was caused by changes in the implantation parameters of the femoral component was plotted by the range analysis of the orthogonal array (Fig. 7). According to the trend diagram, the optimal implantation parameters for the femoral component were those of group A_2_B_1_C_2_, which corresponded to 1° femoral flexion, 5° valgus angle, and 4° external rotation.

### Validation of the optimized parameters derived from orthogonal array testing

In order to validate the optimization results yielded by orthogonal array testing, a finite element model of the TKA knee was constructed based on the optimized implantation parameters, namely A_2_B_1_C_2_. The minimal peak value of the pressure was 16.46 MPa, which was 46.6% lower than the maximal peak value (30.83 MPa) and 9.3% lower than that of the standard model.

## Discussion

The methods that enable accurate implantation of the TKA prosthesis for each patient can ensure that intra-articular stress is evenly distributed across the liner. The minimization of stress would be expected to reduce the wear of the polyethylene liner and prolong the survival of the TKA prosthesis. In the current study, the optimized implantation parameters for the TKA prosthesis were obtained using finite element analysis in combination with orthogonal array testing. The lowest pressure on the polyethylene liner was achieved with femoral component implantation parameters that included 1° flexion, 5° valgus angle, and 4° external rotation. The present findings indicate that among the three factors that influence the pressure on the polyethylene liner, the external rotation angle had the greatest effect followed by the valgus and the flexion angles. This suggests that both the external rotation angle and the valgus angle are important parameters during TKA surgery that are required to ensure accurate implantation of the prosthesis. We envisage that our technique could be used clinically during preoperative planning to optimize the implantation parameters based on the anatomic characteristics of each individual patient.

Although TKA can effectively ameliorate pain and improve the function of the knee joint, the post-TKA satisfaction rate (90%) was reported to be lower than that noted for total hip arthroplasty [34]. Surgical technical error is the most common cause of TKA failure [7]. Improper implantation of a TKA prosthesis leading to loss of the neutral mechanical axis of the lower extremity is a common technical issue, and orthopedic surgeons have attempted various approaches in order to improve the accuracy of implantation, the therapeutic efficacy, and the post-TKA satisfaction. A detailed preoperative planning can aid the optimization of the accuracy of prosthesis implantation and restore the neutral alignment of the mechanical axis. Currently, prosthesis implantation parameters are usually obtained from preoperative imaging studies of the patient’s lower extremities [19]. In the current study, the TKA prosthesis implantation parameters were determined through finite element analysis in combination with orthogonal array testing. This is a technique that has shown great promise in the improvement of the accuracy of prosthesis implantation and the reduction of the surgical technical error. The present study utilized computer simulations that allowed preoperative prediction of the distribution of the intra-articular stress. Using finite element analysis and orthogonal array testing, it was possible to reduce the peak value of the pressure on the polyethylene liner by 9.3% compared with conventional preoperative measurements. Such a reduction in peak pressure would be predicted to reduce the wear of the polyethylene liner and decrease the rate of prosthesis loosening, thereby increasing the survival time of the prosthesis. The current method that was developed for the determination of the prosthesis implantation parameters differs from that used conventionally and has not been described previously. Although the research objectives differed between the present study and previous investigations, the distribution of stress in the standard model of our study was similar to that of models described in the published literature [6, 35, 36]. Moreover, the peak value of the stress in the medial compartment was higher than that in the lateral compartment [6, 35, 36].

Accurate implantation of a prosthesis can lead to a neutral mechanical axis of the lower extremity, longer prosthesis survival, amelioration of pain, and improved function [37, 38]. Previous studies have suggested that a deviation in the mechanical axis of the lower extremity of equal to and/or less than 3° (≤ 3°) can achieve optimal clinical efficacy and longer survival. Huijbregts et al. demonstrated that the prognosis of patients was poor when the deviation of the mechanical axis of the lower extremity was larger than 3° in the coronal plane, implying that the distal femoral osteotomy angle in the coronal plane was an essential factor that contributed to the efficacy of the prosthesis [39]. Kim et al. determined that longer stability could be achieved when the mechanical axis of the femoral component in the sagittal plane ranged between 0° and 3° [40]. In addition, Gromov et al. reported that the clinical results were optimal and the survival period was increased to its maximum when the external rotation angle that was relative to the posterior condylar axis, ranged between 2° and 5° in the axial plane [41]. Consistent with previous investigations, we determined that the optimized implantation of a femoral component could reduce stress in the liner, with the key parameters for accurate implantation being the femoral flexion, the valgus, and external rotation angles. A notable finding of the present study was that the peak value of the pressure on the polyethylene liner varied greatly from a maximal value of 30.83 MPa to a minimal value of 16.46 MPa, corresponding to a difference of 14.37 MPa (87.3%). This variation was evident even within the conventionally accepted range of parameters for the implantation of the femoral component. It was reported by Matsuda et al. that a 5° alteration of the valgus angle in the coronal plane led to a 50% increase in the contact stress on the polyethylene liner of a femoral component [42]. By comparison, we found that a 2° alteration of the valgus angle, from 1° valgus to 1° varus, led to an 87.3% increase in the stress on the polyethylene liner. Moreover, additional alterations in the sagittal plane and in the axial rotation synergistically amplified this effect. Therefore, alterations in the implantation parameters of a femoral component can greatly affect the peak value of the pressure on the polyethylene liner, even when the surgical technical error is within the clinically accepted range. This highlights the potential benefits of developing new techniques in order to refine the accuracy of TKA prosthesis implantation.

Previous studies have shown that neutral mechanical alignment (0° ± 3°) of the lower extremities after TKA surgery can obtain a better long-term prosthesis survival rate [37–39]. However, it is still possible that aseptic loosening can occur after TKA surgery [43]. Lee et al. reported that 13 of 687 TKA knee joints in the neutral position had aseptic loosening within 8 years [44]. In this study, compressive stress in the medial or lateral compartment was not evenly distributed even when the positional alignment of the lower extremities was controlled at 0° ± 1°. The uneven distribution of stress in the medial and lateral compartments may eventually lead to the occurrence of aseptic loosening of TKA knee joints with a neutral mechanical alignment. In 7 of the 10 constructed models of the TKA knee joint, the peak value of the compressive stress was higher in the medial compartment than in the external compartment. Moreover, the highest peak value of compressive stress (30.83 MPa) was in the medial compartment, which is consistent with the clinical phenomenon that the internal tibial plateau of the TKA knee joint is susceptible to bone resorption and collapse [45]. These findings further suggest that we should pay attention to maintaining good rotation, flexion alignment, and soft tissue balance of the prosthesis as well as ensure alignment of the lower extremities in the neutral alignment after TKA surgery.

Previous studies have utilized finite element analyses in order to elucidate the effects of implantation parameters on the stress of the femoral component. These investigations have suggested that different parameters exert varying effects. One study determined that the internal-external rotation, the medial-lateral translation, and the anterior-posterior translation could change the stress on a polyethylene liner by 27.1, 23.3, and 7.63%, respectively [26]. Among these parameters, the angle of the external rotation had the greatest effect [26]. Similarly, it was found by Liau et al. that alterations in the internal translation, internal rotation, and varus angle of a femoral component could induce changes in the stress of 67.6, 14.3, and 145.9%, respectively; the varus angle exerted the largest effect on contact stress [46]. In the current study, the flexion, valgus, and external rotation angles of the TKA prosthesis were analyzed by orthogonal array testing in order to investigate their effects on the pressure on the polyethylene liner. The current method resulted in the determination of the optimal parameters that could be used for preoperative planning and during the surgery in order to achieve accurate implantation. The optimal parameters for femoral component implantation were 1° flexion, 5° valgus angle, and 4° external rotation, which minimized the peak value of the stress on the polyethylene liner to 16.46 MPa. These parameters differed from the parameters obtained by conventional anatomic measurements based on radiography, i.e., 0° flexion, 6° valgus, and 3° external rotation [41]. Using the conventional parameters, the peak value of the pressure on the polyethylene liner was 18.14 MPa (i.e., 9.3% higher than that for the optimal parameters), implying that an implanted femoral component with a little bit of flexion, reduced valgus, and increased external rotation could decrease the stress on the liner. An optimal match between the femoral component and the polyethylene liner was obtained and resulted in a maximum increase in the contact surface area and a minimum decrease in the stress on the liner [26]. In the current study, the variation of the three factors was within the clinically acceptable range and this revealed that the external rotation angle exhibited the greatest effect on the stress, followed by the valgus and the flexion angles. It is likely that the external rotation and the valgus angles exhibited a greater effect on the matching between the femoral component and the polyethylene liner compared with the flexion angle. This indicated that the suboptimal values used for the external rotation and the valgus angles could contribute notably to increased wear damage to the liner. Therefore, during TKA, attention should be paid with regard to the correct external rotation and valgus angles.

A notable feature of the current study was that the orthogonal array testing was utilized in order to reduce the number of TKA models and enhance the efficiency of the analysis. A total of 27 finite element models of the TKA knee would be required in order to include three implantation parameters as well as three different levels in the analysis; the orthogonal array testing effectively reduced the workload to only nine models. Therefore, the orthogonal array testing simplified the preoperative analysis and was far less time-consuming compared with the use of the 27 models. To the best of our knowledge, this is the first study to demonstrate that the combination of finite element analysis and orthogonal array testing can enhance the efficiency of preoperative planning with regard to the optimization of the implantation of the TKA prosthesis. We suggest that this approach could be applied in clinical practice to improve the accuracy of TKA.

The method of femoral component implantation optimization used in this study and the results obtained are not applicable to kinematically aligned TKA because the surgical ideas and principles of mechanically aligned TKA are completely different to those of kinematically aligned TKA. The principle of mechanically aligned TKA surgery is to correct lower extremity deformity and restore the neutral mechanical alignment of the lower extremities (achieved through varying amounts of resection of the medial and lateral condyles and through soft tissue balance, etc.) in order to distribute the load evenly in the knee joint [15, 18]. However, the idea of kinematically aligned TKA is to restore the natural physiological state and mechanical alignment of the lower extremity, using techniques such as equal amounts of resection of the medial and lateral condyles, and to reduce the release of soft tissues as much as possible [47, 48]. For the knee joint in our case, the optimal implantation parameters for the femoral component were 1° flexion, 5° valgus, and 4° external rotation. However, if kinematically aligned TKA was applied, the implantation parameters for the femoral component would need to be 0° flexion (to maintain a good patellar tracking), 8° valgus (through measurement, to ensure equal amounts of resection of the distal femur), and 0° external rotation (to ensure equal amounts of resection of the posterior femoral condyle). Furthermore, kinematically aligned TKA usually uses a cruciate-retaining knee prosthesis rather than a posterior-stabilized knee prosthesis.

The present study has some limitations. Firstly, the cortical and cancellous bones of the knee joint are nonlinear materials, and their properties would be even more complex under pathological conditions such as osteoporosis. However, the bones of the knee joint were simplified into isotropic linear elastic materials. Nonetheless, we believe this simplification is acceptable when the elastic modulus of the femoral and tibial prosthesis is considered. Furthermore, for patients with severe osteoporosis, the elastic moduli of the femur and tibia could be reduced during the process of finite element analysis. Secondly, the study was conducted under the simulated condition of a static, straight position. The results would be more reliable if the intra-articular stress distribution was analyzed under dynamic conditions, for example if the changes in stress in the knee joint were examined under different flexion angles. Thirdly, only the femoral component was optimized, whereas optimization of the tibial component was not considered. Optimization of the tibial component as well as the femoral component would likely yield results that were closer to the clinically accepted targets. Lastly, the emphasis of the study was the accuracy of femoral component implantation and the soft tissue balance was not considered. In future studies, the effect of soft tissue balance on intra-articular stress should be explored.

## Conclusions

In summary, the current study demonstrated that the implantation of a TKA femoral component could be optimized using finite element analysis in combination with orthogonal array testing in order to minimize the peak value of the pressure on the polyethylene liner. Furthermore, the effects of various implantation parameters on the stress were determined. Notably, even minor alterations of the implantation parameters within the clinically acceptable range resulted in substantial changes in the peak value of the pressure on the polyethylene liner. The orthopedic surgeon should pay particular attention to the external rotation and valgus angles in order to ensure accurate implantation of a femoral component. The method based on finite element analysis of femoral component implantation could also be used for tibial component implantation. Our novel technique represents a new approach for preoperative planning and optimization of the implantation parameters in order to achieve the best possible TKA results.

## Abbreviations

3D: Three-dimensional
TKA: Total knee arthroplasty
